# Telehealth Parenting Program and Salivary Epigenetic Biomarkers in Preschool Children With Developmental Delay: NIMHD Social Epigenomics Program

**DOI:** 10.1001/jamanetworkopen.2024.24815

**Published:** 2024-07-29

**Authors:** Sarah M. Merrill, Christina Hogan, Anne K. Bozack, Andres Cardenas, Jonathan S. Comer, Daniel M. Bagner, April Highlander, Justin Parent

**Affiliations:** 1 Department of Psychiatry and Human Behavior, Warren Alpert Medical School, Brown University, Providence, Rhode Island; 2 Department of Psychological and Brain Sciences, University of Massachusetts, Amherst; 3 Department of Epidemiology and Population Health, School of Medicine, Stanford University, Stanford, California; 4 Department of Pediatrics, School of Medicine, Stanford University, Stanford, California; 5 Department of Psychology, Florida International University, Miami; 6 Department of Psychology, University of Rhode Island, Kingston

## Abstract

**Importance:**

Children with developmental delays are at a heightened risk of experiencing mental health challenges, and this risk is exacerbated among racially minoritized children who face disproportionate adversity. Understanding the impact of parenting interventions on biological markers associated with these risks is crucial for mitigating long-term health disparities.

**Objective:**

To examine the effect of 20 weeks of an internet-based parent-child interaction training (iPCIT) program on biomarkers associated with aging and chronic inflammation among preschoolers with developmental delay at 12-month follow-up.

**Design, Setting, and Participants:**

An observational secondary analysis of data from a randomized clinical trial conducted from March 17, 2016, to December 15, 2020, to assess changes in salivary DNA methylation (DNAm)-derived biomarkers following iPCIT intervention. Participants were recruited from 3 Part C early intervention sites in a large southeastern US city. Eligible participants included children recruited within 3 months of their third birthday who had a Child Behavior Checklist Externalizing Problems T score greater than 60 and provided saliva in at least 1 study wave. Data analysis was conducted May 2023 to April 2024.

**Intervention:**

Participants received either iPCIT (a telehealth therapeutic intervention focused on enhancing the parent-child relationship and addressing behavioral challenges in young children) or referrals as usual.

**Main Outcomes and Measures:**

DNAm at the 12-month follow-up was assessed using the Infinium HumanMethylationEPIC Bead Chip Assay to derive biomarkers DunedinPACE, C-reactive protein (CRP), and interleukin-6 (IL-6). Analyses were intent-to-treat and used path analysis.

**Results:**

A total of 71 children (mean [SD] age, 36.27 [0.61] months 51 male [71.8%] and 20 female [28.2%]) were analyzed, of whom 34 received iPCIT and 37 received referrals as usual. The iPCIT group had a slower pace of aging (β = 0.26; 95% CI, 0.06 to 0.50; *P* = .03) and less DNAm-derived CRP (β = 0.27; 95% CI, 0.05 to 0.49; *P* = .01) relative to the control condition at the 12-month follow-up. These associations remained significant after accounting for baseline DNAm score, child demographics, and symptom severity, and were independent of predicted buccal epithelial cell proportion for both DunedinPACE and CRP. There was no association with DNAm-derived IL-6 (β = 0.14; 95% CI, −0.08 to 0.36; *P* = .21).

**Conclusions and Relevance:**

In this study of a parenting intervention, iPCIT, the association of intervention with decreased molecular markers of inflammation and biological aging suggests their potential to modify aspects of the biological embedding of stress. Understanding the systemic biological impact of such interventions offers insights into addressing health disparities and promoting resilience among vulnerable populations.

**Trial Registration:**

ClinicalTrials.gov Identifier: NCT03260816

## Key Points

**Question:**

Does an evidence-based parenting intervention alleviate salivary DNA methylation (DNAm)–derived biomarkers of stress in children with developmental delays?

**Findings:**

In this secondary analysis of 71 children whose families participated in a randomized clinical trial and received either internet-based parent-child interaction therapy or referrals as usual, there was a significantly slower pace of aging and reduced DNAm-derived C-reactive protein among children who received the intervention, but DNAm-derived interleukin-6 did not differ; these findings persisted when accounting for cell type proportion and baseline characteristics.

**Meaning:**

These findings suggest that parenting interventions may modify stress-related biological aging and inflammation, supporting their potential to enhance the long-term health of children with developmental delays.

## Teaser

This secondary analysis of a randomized clinical trial assesses the association of a telehealth parent-child interaction training program with biomarkers associated with aging and chronic inflammation among preschool-aged children with developmental delay.

## Introduction

Young children with developmental delays are a vulnerable population at a substantially heightened risk for future psychopathology and chronic health conditions compared with typically developing children.^1,2,3^ One potential mechanism for the association of developmental delay with later health problems is the heightened and chronic stress these children experience due to cyclical patterns of behavior escalation and reactive parenting. For example, children with developmental delays often exhibit disruptive behaviors that contribute to increases in parental stress, which increases the likelihood of reactive, harsh, or controlling parenting behaviors, thereby engendering a cyclical pattern of chronic stress.^4,5^

This cycle of stress and reactivity is further exacerbated when families already experience heightened levels of adversity.^6,7^ In particular, Black and Latino children are at higher risk for developmental delays compared with non-Hispanic White children^8,9,10,11^ due to disproportionate experiences of social, economic, and environmental adversity, which is further compounded by immigration-, culture-, and racism-related stressors.^8,12,13,14,15,16^ The compounding stressors of family stress related to delays and adversity due to racial minoritization may get under the skin of children with developmental delays,^17^ possibly to the detriment of later-life health.^18^ One mechanism through which this phenomenon may occur is DNA methylation (DNAm), an epigenetic mark that can affect DNA transcription without changing the underlying sequence.^19^ Supporting the idea that changes in DNAm are a mechanism for health disparities in children with developmental delay, research has demonstrated this epigenetic marker to be sensitive to the environment, including parenting^20,21^ and stress,^22^ especially during early childhood.^23^ Because DNAm is critical for establishing and maintaining cell type identity,^19^ differences in cell type proportions can also be informative of inflammation and health in oral tissues,^24,25,26,27,28^ which may be particularly pertinent for those with developmental delays prone to oral bacteria.^29,30,31^

One specific area of DNAm research has found robust evidence for the association of early life stress with biology through the acceleration of DNAm-based pace of biological aging and chronic inflammatory response.^32^ DNA-derived estimates of biological aging can be at pace with chronological aging or can move faster and be accelerated relative to chronological age, which is known as epigenetic age acceleration. Acceleration of epigenetic age in young and later adulthood is associated with morbidity and mortality across many studies.^33,34^ Additionally, greater epigenetic age acceleration has been found with increases in harsh parenting and experiences of threat,^35^ as well as poverty,^36^ minoritized racial identity, parental psychopathology and other disruptions to caregiving,^37,38^ and simultaneous increases in DNAm-derived markers of inflammation.^32,39^ Increases in biomarkers of inflammation have been also been independently associated with harsh parenting, family stress, and later psychopathology.^40,41,42^

Given that early childhood is a particularly sensitive developmental period for the biological embedding of adversity,^23^ early intervention during this stage offers a vital opportunity to mitigate its future consequences. Furthermore, given the clear association of chronic stress with parenting behaviors, interventions that enhance parenting behaviors present a promising avenue. One well-supported and accessible behavioral parenting intervention is internet-based parent-child interaction training (iPCIT),^43^ a telehealth-based therapeutic intervention that enhances the parent-child relationship and address behavioral challenges in young children across cultural contexts.^1,21^ iPCIT focuses on real-time parent-child interactions in children’s natural settings and increased warmth and responsiveness, while reducing harsh and controlling parenting and inconsistent discipline practices.^1^ We previously found that improvements in parenting were associated with decreases in epigenetic age acceleration, but only for children in the most adverse environments.^21^ Therefore, it is still unknown if randomized participation in iPCIT, regardless of individual differences in adversity exposure or parenting improvement, could reduce inflammation and the pace of aging observed in DNAm.

In the current study, we evaluated, via an observational secondary analysis of participants from a randomized clinical trial (RCT) of iPCIT, how enhancing parenting practices alters DNAm-derived biomarkers of pace of aging and inflammation in a sample of predominately Hispanic and Latino preschoolers with developmental delay. We hypothesized that children randomized to the iPCIT treatment condition would demonstrate a reduced DNAm-derived pace of aging and chronic inflammation relative to children randomized to the control condition. Using a longitudinal RCT design, we sought to determine whether family participation in a behavioral parenting intervention (ie, iPCIT) impacts systems of the biological embedding of stress, in turn fostering a biological foundation that promotes resiliency and potentially disrupts detrimental developmental trajectories.

## Methods

### Participants

This secondary analysis of an RCT followed the Consolidated Standards of Reporting Trials (CONSORT) reporting guideline reporting guideline. Participants for the current study were a subsample of the primary RCT^1^ who consented to participate in an optional substudy involving the collection of DNA. For the primary RCT, families were recruited between March 17, 2016, and August 28, 2019, at 3 Part C Early Intervention (EI) sites in a large city in the southeastern US with follow-up through December 15, 2020. Recruitment occurred during the child’s EI exit evaluation within 3 months of the child’s third birthday. Inclusion criteria among these youths with developmental delay were (1) Child Behavior Checklist (CBCL) Externalizing Problems T score greater than 60 and (2) primary caregiver speaks English or Spanish. Exclusion criteria were (1) child receiving medication for behavior problems, (2) child or caregiver deafness or blindness, (3) severe child social communication deficits (ie, caregiver report on Social Responsiveness Scale-Second Edition T score >75), and (4) primary caregiver standard score less than 4 on vocabulary subtest of the Wechsler Abbreviated Scale of Intelligence.^44^ The Florida International University institutional review board approved both the primary RCT and DNA substudy under separate protocols. Written informed consent was provided by parents for themselves and all children in the study. See the study protocol in Supplement 1 for more detailed information on the study.

### Procedure

Eligible families were randomized 1:1 (stratified by child sex) to up to 20 weeks of either iPCIT or referrals as usual (RAU). Major assessments were conducted in the family’s home at baseline, week 20 (posttreatment or RAU), and 6- and 12-month follow-ups. Families received $100 for each major assessment ($50 for midtreatment) and an electronic tablet at study completion. Substudy informed consent was obtained at either baseline, posttreatment, or follow-up home visits, depending on families’ progress in the intervention at the time of institutional review board approval for the DNA substudy. All participants completed the treatment phase before the COVID-19 pandemic.

### Parenting Intervention

iPCIT uses encrypted videoconferencing technology through which therapists remotely provide live coaching of caregiver-child interactions in their own homes.^43^ As in clinic-based PCIT, iPCIT progresses through child-directed interaction and parent-directed interaction phases. During child-directed interaction, caregivers learn to follow their child’s lead in play by using praise and child-focused attention and avoiding questions, commands, and criticisms.^43^ They learn to use positive parenting skills in response to appropriate child behaviors and to actively ignore undesirable behaviors. During parent-directed interaction, caregivers learn to use effective commands and consistently follow through with timeouts to increase child compliance. iPCIT treatment was offered in English and Spanish. Families randomized to RAU were referred by their previous EI clinician to community-based treatment services, as necessary. The primary RCT^1^ found that iPCIT led to improvements in child behavior and increases in warmth and responsiveness maintained across follow-up visits relative to the control condition.

### Salivary Sample Collection

At all home visits, saliva DNA samples were collected from children using Oragene kits (OGR-575) for assisted collections (DNA Genotek). DNA was extracted and isolated using the DNEasy extraction system (Qiagen) and assessed for quantity and quality using a Nanodrop spectrophotometer. Recent evidence supports the validity of this noninvasive DNA collection technique for DNAm.^45,46^

### Microarray Quantification of DNA Methylation

We measured DNAm using established protocols at the University of Minnesota Genomics Center and DNA Genotek. Genomic DNA was bisulfite converted and quantified on the Infinium HumanMethylationEPIC Bead Chip Assay and processed with R version 4.1.1 (R Project for Statistical Computing)^47^ packages minfi^48^ and ewastools,^49^ with samples with median intensity values excluded. Normalization was performed with the packages funnorm with noob for background and color adjustment.^50^ Buccal epithelial cell (BEC) proportion was estimated using EpiDISH in R.^51^

### Covariate Measures

The CBCL for ages 1.5 to 5 years is a 99-item caregiver-rating scale that measures the frequency of children’s behavioral and emotional problems, showing excellent psychometric properties,^52,53^ including for children with developmental delay.^1,54^ The Total Problems raw score was used to measure caregiver-reported child total baseline symptom severity. The total score of the Depression, Anxiety, and Stress Scale^55,56^ was used to assess parental stress and psychopathology.

### Calculation of Biological Age Acceleration

The DunedinPACE pace of aging,^57^ previously employed in pediatric saliva samples,^32,39^ was the epigenetic aging outcome in the current study. Pace of aging was calculated using the publicly available function in R.^57,58^ A value of 1 in this measure indicates the estimated epigenetic age and chronological age were equivalent, with a greater value indicating epigenetic age acceleration in comparison with chronological age. In this pediatric sample, the pace of aging measure was slightly skewed toward increased epigenetic age acceleration with a range from 0.98 to 1.56.

### Calculation of DNA Methylation-Predicted Stress Biomarkers

A C-Reactive Protein (CRP)—a protein released by the liver in response to inflammation throughout the body—DNAm risk score was estimated using the sum of coefficient weights of a previously published trans-ethnic discovery analysis.^59^ This score was previously applied in pediatric salivary DNAm, in which predicted CRP differences were sensitive to children’s parent-reported internalizing symptoms and aggression problems^39^ and has been found in adult populations to be more indicative of chronic inflammation than circulating CRP.^60^ Interleukin-6 (IL-6) was estimated with DNAm using previously described methods.^61^

### Statistical Analysis

The current DNAm substudy began after the primary RCT began, resulting in the current study being an observational, nonrandomized, secondary analysis of participants in the iPCIT trial. This resulted in a larger portion of missing data at baseline than at the final follow-up. In the current study, participants were included if they had DNAm that passed control thresholds for at least 1 time point. As part of an inclusive analysis strategy to optimize the missing data model,^62^ we included race and ethnicity, parental stress, parent primary language, and all available waves of DNAm of the model outcomes as auxiliary variables. Race and ethnicity categories included non-Hispanic Asian, non-Hispanic Black or African American, Black Latino or Hispanic, Latino or Hispanic, and non-Hispanic White. Analysis of variance and χ^2^ tests were used to assess differences between the families who completed the substudy with all families from the original RCT.

Primary models used path analysis and maximum likelihood estimation with robust standard errors in Mplus version 8.8 (Muthén & Muthén). All models included baseline levels of DNAm biomarkers to account for stability and control for any baseline differences between groups. Furthermore, covariates in all models, separate from missing data auxiliary variables, included child sex, race and ethnicity, and baseline CBCL Total Problem severity score. Secondary analyses simultaneously examined changes in DNAm biomarkers (eg, DunedinPACE) and BEC proportion to examine unique associations (see the Figure for the model). Finally, full information maximum likelihood estimation techniques were used for the inclusion of all available data, based on intent-to-treat guidelines. The threshold for statistical significance was a 2-sided *P* < .05. Data analysis occurred from May 2023 to April 2024.

**Figure.**
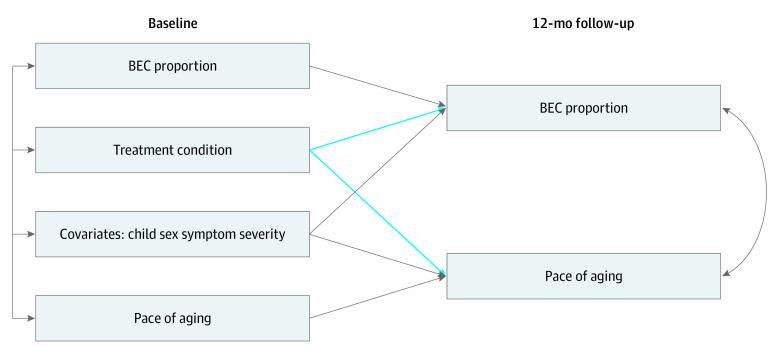
Path Models for Primary Outcomes and Cell Type Covariates are shown as a single observed variable for figure clarity but are separate variables in models. Separate models were run for pace of aging, C-reactive protein, and interleukin-6. BEC indicates buccal epithelial cell.

## Results

### Participants

A total of 162 samples from 71 children (mean [SD] age, 36.27 [0.61] months]; 51 male [71.8%] and 20 female [28.2%]; 3 non-Hispanic Asian [4.2%]; 3 Black Latino or Hispanic [4.2%]; 13 non-Hispanic Black or African American [18.3%]; 48 Hispanic or Latino [67.6%]; 4 non-Hispanic White [5.6%]) were included for analysis in the study; of the 71 children, 34 received iPCIT and 37 received RAU. Of the 34 families who received iPCIT, 16 (48.0%) received treatment in Spanish. Missing data for the 71 families that consented to the DNAm substudy included 39 families (54.9%) at baseline (due to the DNA substudy starting after the primary trial started), 26 families (36.6%) at the 9-month follow-up, and 12 families (16.9%) at the 12-month follow-up. Based on income-to-needs ratio, 44 of 69 families reporting income (63.8%) were classified as in the extreme poverty to low-income categories. See Table 1 for complete demographic details. At baseline, we found that child demographics of the 71 families included in the current study did not significantly differ from the 79 families who did not participate in the DNA substudy; there were also no significant child demographic differences between families who received iPCIT and RAU. Furthermore, participants included in the current study did not significantly differ from those who did not participate in the DNA substudy on posttreatment child symptom outcomes or clinically significant improvement based on the CBCL. Thus, support for data being missing at random was found.

**Table 1.** Study Demographics by Group

Characteristic	Treatment condition, No. (%) (N = 150)^a^			
	Total DNA (n = 71)	iPCIT (n = 34)	RAU (n = 37)	Total non-DNA (n = 79)
Child sex				
Male	51 (71.8)	25 (73.5)	26 (70.3)	60 (75.9)
Female	20 (28.2)	9 (26.5)	11 (29.7)	19 (24.1)
Child age, mean (SD), mo	36.27 (0.61)	36.27 (0.67)	36.27 (0.56)	36.42 (1.2)
Child ethnicity and race				
Non-Hispanic Asian	3 (4.2)	1 (2.9)	2 (5.4)	2 (2.5)
Non-Hispanic Black or African American	13 (18.3)	4 (11.8)	9 (24.3)	15 (19.0)
Black Latino or Hispanic	3 (4.2)	1 (2.9)	2 (5.4)	7 (8.9)
White Latino or Hispanic	48 (67.6)	27 (79.4)	21 (56.8)	49 (62.0)
Non-Hispanic White	4 (5.6)	1 (2.9)	3 (8.1)	6 (7.6)
Primary caregiver age, mean (SD), y	33.13 (7.00)	33.06 (5.65)	33.19 (5.12)	34.87 (7.00)
Primary caregiver gender				
Women	67 (94.4)	33 (97.1)	34 (91.9)	72 (91.1)
Men	4 (5.6)	1 (2.9)	3 (8.1)	7 (8.9)
Primary caregiver education				
Did not complete high school	7 (9.9)	4 (8.8)	3 (8.1)	7 (8.9)
High school graduate	34 (47.9)	17 (50.0)	17 (45.9)	26 (32.9)
College graduate	24 (33.8)	8 (23.5)	16 (43.2)	25 (31.6)
Graduate degree	6 (8.5)	5 (14.7)	1 (2.7)	17 (21.5)
Did not report	NA	NA	NA	4 (5.1)
Primary caregiver marital status				
Married	45 (63.4)	19 (55.9)	26 (70.3)	40 (50.6)
Separated or divorced	4 (5.6)	2 (5.9)	2 (5.4)	11 (13.9)
Single, never married	21 (29.6)	13 (38.2)	8 (21.6)	21 (26.6)
Cohabitating partner	1 (1.4)	0	1 (2.7)	3 (3.8)
Not reported	0	0	0	4 (5.1)
Primary caregiver preferred language				
English	40 (56.3)	17 (50)	26 (70.3)	53 (67.1)
Spanish	28 (39.4)	17 (50)	11 (29.7)	26 (32.9)
Child Behavior Checklist Total T score, mean (SD)	62.6 (11.2)	61.6 (10.9)	63.6 (11.4)	60.6 (10.1)

Abbreviations: iPCIT, internet-based parent-child interaction training; NA, not applicable; RAU, referrals as usual.

^a^ No significant differences between iPCIT and RAU or between DNA and non-DNA were found.

### Primary Analyses

See Table 2 for complete results. For pace of aging (DunedinPACE) at the 12-month follow-up, statistically significant treatment differences were observed after accounting for baseline DunedinPACE, biological sex, race and ethnicity, and baseline child symptom severity. The iPCIT group showed a slower pace of aging relative to the RAU control condition (pace, 1.27 vs 1.32) with a medium effect size (Cohen *d* = 0.55). In secondary analyses, we simultaneously examined estimated BEC proportion at baseline and 12-month follow-up along with DunedinPACE. The pace of aging and BEC proportion were highly correlated at follow-up (β = .61; 95% CI, 0.41-0.81; *P* < .001), and significant treatment condition associations were observed (β = 0.26; 95% CI, 0.03-0.49; *P* = .03). The significant association of treatment with the pace of aging remained with a similar effect size after accounting for the covariation between DunedinPACE and BEC proportion (β = 0.29; 95% CI, 0.05-0.52; *P* = .02).

**Table 2.** Primary Results for Each Outcome

Models and Outcomes^a^	B (95 CI)	β (95% CI)	*P* value
Dunedin PACE			
Baseline Dunedin PACE	0.19 (−0.37 to 0.75)	0.23 (−0.47 to 0.91)	.53
Child sex^b^	−0.05 (−0.11 to −0.01)	−0.29 (−0.57 to −0.01)	.04
Race^c^	0.01 (−0.05 to 0.05)	0.01 (−0.25 to 0.22)	.96
Baseline severity^d^	−0.01 (0.00 to 0.00)	−0.17 (−0.42 to 0.08)	.18
Treatment^e^	0.05 (0.01 to 0.09)	0.26 (0.03 to 0.50)	.03
CRP			
Baseline CRP	0.04 (−0.44 to 0.53)	0.04 (−0.42 to 0.51)	.86
Child sex^b^	−22.99 (−59.75 to 13.77)	−0.17 (−0.43 to 0.10)	.22
Race^c^	−9.22 (−41.89 to 23.44)	−0.06 (−0.28 to 0.12)	.58
Baseline severity^d^	−0.72 (−1.25 to −0.20)	−0.31 (−0.53 to −0.09)	.006
Treatment^e^	34.12 (5.19 to 63.05)	0.27 (0.05 to 0.49)	.01
IL-6			
Baseline IL-6	0.46 (0.14 to 0.78)	0.58 (0.17 to 0.99)	.006
Child sex^b^	−0.01 (−0.01 to 0.04)	−0.07 (−0.29 to 0.15)	.53
Race^c^	−0.01 (−.05 to 0.03)	−0.08 (−0.45 to 0.28)	.65
Baseline severity^d^	−0.00 (−0.00 to 0.00)	−0.38 (−0.62 to −0.14)	.002
Treatment^e^	0.01 (−0.01 to 0.04)	0.14 (−0.08 to 0.36)	.21

Abbreviations: CRP, C-reactive protein; IL-6, interleukin 6.

^a^ Each outcome model is estimated separately.

^b^ Child sex was coded as 1 = male, 2 = female.

^c^ Race was coded for analyses as a binary variable with 1 = Black or African American.

^d^ Baseline severity was based on the Child Behavior Checklist total raw score.

^e^ Treatment was coded as 1 = internet-based parent-child interaction training and 2 = referrals as usual.

For DNAm-derived CRP at the 12-month follow-up, statistically significant treatment differences were observed after accounting for baseline CRP and other covariates. The iPCIT group showed lower mean levels of DNAm-derived CRP relative to the control RAU condition (84.01 vs 117.04) with a medium effect size (*d* = 0.58). Compared with the RAU group, the iPCIT group had less DNAm-derived CRP (β = 0.27; 95% CI, 0.05 to 0.49; P = .01) relative to the control condition at the 12-month follow-up. In secondary analyses, the significant association of treatment with CRP remained after the inclusion of cell type in the model (β = 0.31; 95% CI, 0.03 to 0.53; *P* = .005). Lastly, for DNAm-derived IL-6 at the 12-month follow-up, there were no statistically significant treatment differences between those who received iPCIT and RAU (mean IL-6, 0.57 vs 0.58) and there was no association with DNAm-derived IL-6 (β = 0.14; 95% CI, −0.08 to 0.36; *P* = .21). Upon residualizing for predicted BEC proportion, DNAm-derived IL-6 was weakly positively correlated with DNAm-derived CRP (*r* = 0.30; *P* = .02), but strongly positively correlated with DunedinPACE (*r* = 0.75; *P* < .001). However, DunedinPACE was moderately positively correlated with DNAm-derived CRP in the sample (*r* = 0.41; *P* = .001). In secondary analyses, we examined a model with both CRP and PACE as simultaneous outcomes, accounting for their residual covariance (7 CpG sites were overlapping between measures). In this model, the statistically significant association of treatment remained with CRP (β = 0.28; 95% CI, 0.05 to 0.51; *P* = .02). However, in this model, the association of treatment with DunedinPACE was attenuated (β = 0.20; 95% CI, −0.01 to 0.42; *P* = .07), suggesting part of the association of treatment with the pace of aging may be driven by shared variance with DNA-based CRP.

## Discussion

In our secondary analysis of an RCT, we observed the association of an evidenced-based parenting intervention, iPCIT, with slowing the pace of aging and reducing DNAm-derived CRP. We did not observe an association with DNAm-derived IL-6. Furthermore, the association of iPCIT participation with decreased pace of aging and CRP was independent of estimated cell composition. The effect sizes observed in this study for DNAm-derived CRP were similar to the associations found with lower brain volume in preterm infants^63^ and adults^64^ and larger than the association with lower cognitive ability in older adults.^65^ Both the observed medium effect sizes of iPCIT for DNAm-derived CRP and pace of aging were larger than previously reported cross-sectional associations of these measures in pediatric saliva with parent-reported higher internalizing symptoms.^39^ Additionally, the effect size of intervention on pace of aging alone was larger than that of parent-reported higher aggression^39^ and living in more disadvantaged neighborhoods^32^ in pediatric saliva, while being similar to a caloric restriction intervention in adult blood.^66^ Together, these results indicate the potential for the extent of changes observed in these epigenetic biomarkers in response to a parenting intervention to associate with clinically relevant outcomes.

The positive impact of iPCIT on the rate of epigenetic aging suggests parenting interventions may have the potential to modify aspects of stress-related biological embedding. The pace of aging, by design, is rooted in the dynamics of health and phenotypes supporting successful biological aging processes.^34,57^ In a previous study, we found no main treatment association with a different epigenetic age marker (PedBE), only an association of parenting among those relatively higher in baseline adversity.^21^ As such, it may be that because DunedinPACE encompassed multiple biological domains of health in its development,^57^ this measure may reflect systemic biological effects on processes related to stress and coping.

CRP is an inflammatory response protein^67^ believed to elevate in the context of chronic stress.^68^ Recent research indicates DNAm-derived CRP is longitudinally stable and may be more reflective of sustained inflammation than serum CRP, especially regarding associations with neural outcomes.^69^ The observed decrease in DNAm-derived CRP for participants who received iPCIT aligned with established literature associating psychosocial stressors with CRP inflammatory responses^68^ and further supported the interpretation of these findings as potential evidence for sensitivity within a more systemic biological response to stressors. Parenting interventions, by fostering supportive and nurturing environments, might mitigate chronic stressors that contribute to elevated CRP levels. However, more research is needed as to the systemic nature and health implications of these findings.

The lack of an association with DNAm-derived IL-6, an inflammatory cytokine that stimulates CRP production, may be attributed to the multifaceted nature of the inflammatory response.^70^ Unlike CRP, IL-6 is produced by multiple cells and, while referred to as a proinflammatory cytokine, serves both proinflammatory and anti-inflammatory roles in different contexts.^70^ The focus of iPCIT on parenting behaviors may not have been sufficient to induce changes in the more complex and upstream IL-6 regulatory pathways, or these changes may have been contradictory across systems, resulting in no observable change based on our DNAm prediction.

### Limitations

The findings in the current study need to be understood considering its limitations. The tissue measured for this study was saliva, a noninvasive sample with the benefit of containing both BEC, deriving from the same germ layer as brain,^46^ and immune cells activated in response to inflammation.^71^ Additionally, although the study benefited from conducting a longitudinal secondary analysis of RCT participants for better causal inferences, the sample size for this study was small and largely missing DNAm baseline data. Due to this sampling issue, we did not examine trajectories of DNAm across all waves, although this is an important question in the field for future research. Furthermore, participants consented to the DNAm substudy at various waves, sometimes after randomization occurred, which resulted in the study being observational which calls into question randomization and being able to make strong causal inferences.

## Conclusions

In conclusion, the observed reduction in the DNAm-derived pace of aging and CRP, coupled with the absence of associations with IL-6 and PedBE age acceleration,^21^ underscores the intricate association of parenting interventions with biological markers of aging and inflammation. The specificity of iPCIT intervention effects underscores the necessity of targeted interventions tailored to address distinct aspects of the complex biological and behavioral landscape. Future research could deeply phenotype the molecular mechanisms involved and explore the long-term implications of interventions on later health, development, and well-being. Furthermore, future research should explore if intervention-related changes are enduring across development and reduce the long-term consequences of health disparities.

## Notes

Supplement 1.
Trial Protocol

Supplement 2.
Data Sharing Statement
