# Supplementary material for: Telehealth Parenting Program and Salivary Epigenetic Biomarkers in Preschool Children With Developmental Delay: NIMHD Social Epigenomics Program
Source: JAMA Netw Open. 2024 Jul 29;7(7):e2424815. doi: 10.1001/jamanetworkopen.2024.24815 (PMC11287424; doi:10.1001/jamanetworkopen.2024.24815)
Supplement: Supplement 1. — Trial Protocol [file jamanetwopen-e2424815-s001.pdf]

**Supplement 1**: Extended Protocol for IRB-15-0134, Dr. Daniel M. Bagner

# Advancing Child Competencies by Extending Supported Services (ACCESS) for Families Program

## 1. Purpose

The purpose of this research study is to evaluate, via a randomized controlled trial, the effects of a telehealth format of Parent-Child Interaction Therapy (i.e., Internet-delivered PCIT, or iPCIT) for 3-year-old children with developmental delay (DD) and behavior problems. Specifically, this study will examine the impact of iPCIT on child disruptive behavior problems, caregiver behaviors, and caregiver stress relative to traditional referrals as usual (RAU), among youth aging out of Part C Early Intervention (EI) services. Secondary goals of this study are to examine the impact of iPCIT on additional child outcomes (e.g., child pre-academic skills) and to evaluate mechanisms that explain under which circumstances, for whom, and through which pathways iPCIT is most effective for young children with DD.

Investigators hope to learn the effects of iPCIT on young children with behavior problems who are aging out of Part C EI services, relative to RAU. Early behavior problems in children with DD are prevalent and impairing. Despite support for parenting interventions, barriers to care (e.g., transportation obstacles, provider shortages, limited availability of non-English language services, and stigma-related concerns) persist. This study will provide information on the utility of Internet-delivered treatment, which may expand the reach and scope of care for underserved children with DD and their families.

## 2. Study Procedures

The study procedure is as follows:

**Participants:**
Child participants will be at least 3 years of age at baseline. We anticipate recruiting and consenting at least 600 families to enroll 150 eligible families that will complete the full study protocol. The research staff will obtain informed consent from the child's caregiver before any study procedures begin. Consent will be obtained either in-person or over the phone.

**Recruitment and Screening**:
Families will be recruited from three sites: 1) the University of Miami Early Steps office that serves north Miami-Dade County and draws a majority of referrals from high poverty urban neighborhoods, 2) the Nicklaus Children's Hospital Early Steps office that serves south Miami-Dade County and Monroe county (including all of the Florida Keys—a geographically remote and underserved region in South Florida), and 3) the Wingate Oaks Center School in Broward County, the site at which the Children's Diagnostic and Treatment Center's Early Steps office conducts their exit-interviews and transition meetings with families. Exit interviews occur on Wednesdays and the third Thursday of every month at the University of Miami site; on Fridays at the Nicklaus Children's Hospital site; and on Mondays, Tuesdays, Wednesdays, and Thursdays at the Wingate Oaks Center School site.

On exit-interview days, study staff will meet with families at these sites about their potential involvement in the study. Participant recruitment brochures (in English and Spanish) will be available at each recruitment site for participants to read a basic description of the study and conveniently obtain study contact information. Included on the recruitment brochures will be a website link (accessprogram.fiu.edu) to the study website. The study website page includes the same information provided on the brochures with additional information regarding study staff. Study staff will review study procedures with families, and interested families will provide informed consent.

We will end recruitment once we meet our targeted enrollment goal.

**Intervention Procedures:**
After providing informed consent, families will provide their contact information. Afterward, families will complete the screening measures (described in the Measures section below). Families that do not meet remaining inclusion/exclusion criteria after the screening will be given feedback on the results of the screening and offered referrals to alternative services. Families that meet all inclusion and exclusion criteria will be scheduled for a baseline assessment in their home. Eligible families will complete the initial baseline assessment in two formats: (a) completion of caregiver-report questionnaires via hardcopy forms or electronic forms on REDCap, a browser-based electronic data capture platform, and (b) study staff traveling to participant homes to conduct evaluations regarding: caregiver-child interaction observation, child pre-academic skills testing, child cognition and language testing, child height and weight data collection, and child emotion regulation observation. At the conclusion of the home-based assessment, children will select a small toy and caregivers will be compensated $100 in the form of gift cards for their participation. Families will then be randomly assigned to the study conditions of iPCIT or RAU.

**Intervention Delivery:**
The iPCIT draws on real-time, interactive videoconferencing technology to provide live and evidence-based coaching of caregiver-child interactions directly in a family's home. As in standard PCIT, iPCIT consists of roughly 13 to 16 one-hour weekly sessions (precise treatment length is "titrated" with the quickness with which a family reaches skills criteria). Although the delivery format differs from traditional PCIT, the contents of iPCIT directly follow the standard protocol, with the minor exception that the initial session also includes an introduction to technological aspects of treatment. Similar to traditional PCIT (which has the therapist coaching from behind a one-way mirror), iPCIT uses real-time videoconferencing for sessions, delivering live "bug-in-the-ear" coaching to families in their homes. Families will broadcast caregiver-child interactions live from their home via webcam over an encrypted connection, and therapists will provide instantaneous feedback from a remote site. Also, whereas handouts are distributed in traditional PCIT at the end of each session, iPCIT session handouts will be made available online and reviewed in real time with therapists using "share desktop" functions.

Families randomized to iPCIT will receive an equipment package including: 1 iPad Mini (with camera, Bluetooth connectivity, internet access, plastic cover, and stand), 1 Bluetooth speaker/microphone, and 1 Bluetooth headset. For families without household Wi-Fi in their

home, we will provide an Internet plan during the course of treatment. Study staff will contact families over the phone to give the families step-by-step instructions on how to use their materials and join their secure videoconferencing sessions.

After receiving the equipment package and step-by-step instructions, families assigned to the iPCIT group will receive weekly sessions (12- 20 weeks of treatment). Caregivers will wear cordless headsets to afford mobility. Families will complete a weekly pre-session Eyberg Child Behavior Inventory (ECBI) and PCIT homework forms.

**Follow-Up:**
Observational and caregiver-report measures of child and caregiver behaviors and caregiving stress will be examined at Week 0 (pre-treatment/RAU), Week 8 (mid-treatment/RAU), Week 21 (post-treatment/RAU), Week 42 (6 months post-treatment/RAU), and Week 68 (12 months post-treatment/RAU). At each major assessment (Weeks 0, 21, 42, and 68), families will complete all questionnaires
remotely via REDCap (or by paper and pencil if the family prefers) and study staff will complete other assessment procedures (e.g., caregiver-child interaction observation, child pre-academic skills testing, child cognition and language
testing, child height and weight data collection, and child emotion regulation observation) in each family's home either in-person or over a recorded videoconference when necessary. An abbreviated caregiver-report assessment will be collected at Week 8 across conditions. Families will receive a $50 gift card for completion of the Week 8 assessment, $100 gift cards for each major assessment (Weeks 0, 21, 42, 68) and a tablet at study completion.

**Conclusion:**
The final follow-up will take place at Week 68. Families will complete study questionnaires and a study staff member will conduct other assessment procedures either in the family's home or over videoconference if necessary. After completion of the final follow-up, families will receive the final $100 gift card, a tablet (if they had completed all other major assessments as well), and a list of referrals to additional services.

**Measures:**
<u>Screening Measures</u>: The screening will consist of the externalizing scale of the Child Behavior Checklist (CBCL), the Social Responsiveness Scale – Second Edition (SRS-2), and the vocabulary subtest of the Wechsler Abbreviated Scale of Intelligence – Second Edition (WASI-II; for English speakers) or Escala de Inteligencia Wechsler Para Adultos – Third Edition (EIWA-III; for Spanish speakers). Information also collected at the EI site/at the point of consent will be: family demographic information, child height and weight, and the comprehensive results of the Battelle Developmental Inventory (BDI; an evaluation conducted by EI staff members at the child's EI exit interview). After the screening forms are completed, families will be provided with the demographic questionnaire.

<u>Caregiver-rated questionnaires (Weeks 0, 8, 21, 42, 68):</u> Parental Attitudes Toward Psychological Services Inventory (PATPSI); Abbreviated Multidimensional Acculturation Scale (AMAS); Experiences with Technology Survey 2.1 (ETS 2.1); Technological Ease and Computer-based Habits Inventory (TECHI); Child Experiences with Technology

Survey; Beliefs and Attitudes about Technology-based Child Health Resources (BATCH-R); Services Addendum; CBCL; Eyberg Child Behavior Inventory (ECBI); Multidimensional Assessment of Preschool Disruptive Behavior (MAP-DB) temper loss scale; Child Rearing Inventory (CRI); Parenting Practices Inventory (PPI); Family Impact Questionnaire (FIQ); Depression, Anxiety, Stress Scale (DASS-21); Children's Sleep Habits Questionnaire (CSHQ); Technology Experience and Attitude Rating Scale (TEARS); Services for Children/Adolescents – Parent Interview (SCAPI).

Caregiver-rated questionnaires (Week 8 and Week 21 only): Telepresence in Videoconferencing Scale; Barriers to Treatment Participation Scale (BTPS).

Caregiver-rated questionnaires (Week 21 only): Therapy Attitude Inventory; Client Satisfaction Questionnaire-8 (CSQ-8).

Caregiver-rated questionnaires (only parents in the iPCIT condition): Everyday Stressors Index (ESI); Child-Directed Interaction (CDI) Homework Sheet; Parent-Directed Interaction (PDI) Homework Sheet.

Assessor-rated measures (Weeks 0, 21, 42, 68): Bracken School Readiness Assessment – Third Edition (BSRA-3); Preschool Language Scales – Fifth Edition (PLS-5); Dyadic Parent-Child Interaction Coding System – Fourth Edition (DPICS-IV); Impossibility Perfect Circle (IPC) Task; observation of caregiver and child book-reading procedure; and Body Mass Index (BMI) of child.

Teacher-rated questionnaires (when applicable; Weeks 0, 8, 21, 42, 68): Sutter-Eyberg Student Behavior Inventory-Revised (SESBI-R).

iPCIT therapist-rated questionnaires: Clinical Global Impression (CGI-I); TECHI – Therapist Version; Treatment Session Progress Notes; TEARS – Therapist Version.

## 3. Eligibility Criteria

**Inclusion Criteria:**

1. The child is aging out of Part C EI services and at least 3-years-old.
2. The Child Behavior Checklist (CBCL) externalizing problems T-Score is greater than or equal to 60.
3. The child's primary caregiver speaks English or Spanish.

**Exclusion Criteria:**

1. The child is receiving medication for behavior problems.
2. The child or caregiver has a severe physical impairment (e.g., deafness or blindness).
3. The child has severe social communication deficits, (i.e., SRS-2 T-Score is greater than 75).
4. The child's primary caregiver has a standard score less than 4 on the vocabulary subtest of WASI-II (for English speakers) or EIWA-III (for Spanish speakers).

## 4. Outcome Measures & Statistical Methods

**Primary Outcome Measures:**

1. The immediate and one-year impact of iPCIT on disruptive behavior problems in young children with DD (change in CBCL T-score and observed compliance on DPICS-IV).

**Secondary Outcome Measures:**

1. Parenting practices (changes in DPICS-IV and PPI measures), and parental distress in parents of young children with DD (changes in the FIQ measure).
2. Family retention, engagement, and satisfaction associated with iPCIT in young children with DD (TAI and CSQ-8 measures post-treatment).
3. The extent to which technological literacy and access moderate iPCIT efficacy, such that iPCIT efficacy will be weaker among families with poorer technological literacy and/or access.
4. The extent to which traditional barriers to care moderate iPCIT efficacy, such that the incremental efficacy of iPCIT over RAU will be strongest among families with geographic, transportation and/or childcare obstacles to in-person services.

**Other Outcome Measures:**

1. The impact of iPCIT on pre-academic skills among young children with DD (changes in PLS-5 and Bracken scores).
2. The extent to which iPCIT will yield changes in child behavior and pre-academic skills indirectly through direct effects on parenting practices (specifically, changes in parental consistency, warmth, follow-through, and effective discipline).

**Statistical Analysis Plan:**

Analyses will be conducted in SPSS 20 or Mplus 6 using latent growth models within a structural equation modeling (SEM) framework. Baseline and other variables correlating significantly with outcomes will be identified as potential covariates and included in all analyses.

Behavior outcomes will be parent report of disruptive behavior problems and observed percent of child compliance. Parenting practices will be drawn from structured observations and parent report. Parental distress outcomes will be drawn from parent reports. Latent growth of behavior problems, parenting practices, and parental distress will be developed to evaluate change in these constructs over the 5 time points (Weeks 0, 8, 16, 42, and 68). Linear and quadratic effects will be evaluated in both models. A single model in which intervention group predicts the latent slopes of all outcomes will be estimated and evaluated. We expect non-significant changes for the RAU group; we expect the intervention group to show decreases (i.e., negative slopes) in behavior problems, negative parenting practices, and parental distress, and increases (i.e., positive slopes) in child compliance and positive parenting practices. Although pilot study analyses suggest non-linear changes using *t*-tests

(i.e., large initial differences followed by maintenance), preliminary growth model analysis suggests a linear trend alone fits the data better. We also will evaluate a quadratic trend (and associated group differences in the quadratic trend). Compared to RAU youth, we also predict iPCIT youth will exhibit greater early literacy and numeracy skills across time. Latent growth of early literacy and numeracy skills will be developed to evaluate change over the 5 time points (Weeks 0, 8, 16, 42, and 68). Linear and quadratic effects will be evaluated. We expect both groups will show increases (i.e., positive slopes) in literacy and numeracy skills, with steepest slopes found among iPCIT youth.

Chi square analyses will be used to examine whether iPCIT families show higher rates of initiating a first and second treatment appointment than RAU families. We predict at least 70% of iPCIT families will complete a full course of treatment, and we predict therapists will rate iPCIT as a relatively easy protocol to implement at the conclusion of at least 70% of iPCIT sessions. T-tests will examine our prediction that iPCIT parents will report greater satisfaction with services received than RAU families.

We predict that outcomes across time will be significantly greater for families who are high in technological literacy and low in traditional barriers to care. The moderating effects of technological literacy and barriers to care will be evaluated using interaction terms between treatment condition and the moderator to predict outcomes. Compared to RAU families, we predict that the higher levels of positive parenting practices and lower levels of negative parenting practices among iPCIT parents at post-intervention will in turn predict lower levels of child behavior problems and pre-academic skills across time. The effect of group condition on change (i.e., slope) in child behavior problems and pre- academic skills via parenting practices will be evaluated using a joint significance of the indirect effect.

We will minimize missing data through careful training and oversight. Staff will track any data entry issue in REDCap's Data Resolution Workflow and its disposition at the datum level. We will assess missing data mechanisms, such as missing at random (MAR) versus missing not at random (MNAR). The proposed analysis (i.e., latent growth models in an SEM framework) uses all available observations, therefore minimizing the impact of attrition. In addition, SEMs utilize modern missing data approaches such as full information maximum likelihood for data that are missing at random, further minimizing impact of attrition and missing values. If there is evidence for MNAR patterns, we will use appropriate methods.
