# Supplementary material for: Telehealth Parenting Program and Salivary Epigenetic Biomarkers in Preschool Children With Developmental Delay: NIMHD Social Epigenomics Program
Source: JAMA Netw Open. 2024 Jul 29;7(7):e2424815. doi: 10.1001/jamanetworkopen.2024.24815 (PMC11287424; doi:10.1001/jamanetworkopen.2024.24815)
Supplement: Supplement 2. — Data Sharing Statement [file jamanetwopen-e2424815-s002.pdf]

Data Sharing Statement

Merrill. Telehealth Parenting Program and Salivary Epigenetic Biomarkers in Preschool Children With Developmental Delay. *JAMA Netw Open*. Published July 29, 2024. doi:10.1001/jamanetworkopen.2024.24815

Data
Data available: Yes
Data types: Deidentified participant data
How to access data: justin.parent@uri.edu
When available: With publication

Supporting Documents
Document types: Statistical/analytic code
How to access documents: justin.parent@uri.edu
When available: With publication

Additional Information
Who can access the data: anyone requesting the data
Types of analyses: For confirmation or replication of results or pre-approved analysis via a data-sharing agreement
Mechanisms of data availability: With a signed data access agreement
